# Reproduction and the Early Development of Vertebrates in Space: Problems, Results, Opportunities

**DOI:** 10.3390/life11020109

**Published:** 2021-01-31

**Authors:** Alexandra Proshchina, Victoria Gulimova, Anastasia Kharlamova, Yuliya Krivova, Nadezhda Besova, Rustam Berdiev, Sergey Saveliev

**Affiliations:** 1 Research Institute of Human Morphology, Ministry of Science and Higher Education RF, Tsurupi Street, 3, 117418 Moscow, Russia; gulimova@yandex.ru (V.G.); grossulyar@gmail.com (A.K.); homulkina@rambler.ru (Y.K.); brainmicroscopy@yandex.ru (N.B.); braincase@yandex.ru (S.S.); 2 Research and Educational Center for Wild Animal Rehabilitation, Faculty of Biology, M.V. Lomonosov Moscow State University, Leninskie Gory, 1/12, 119899 Moscow, Russia; rberdiev@gmail.com

**Keywords:** development, reproduction, vertebrates, microgravity, space flight, nervous system, fishes, amphibians, reptiles, birds, mammals

## Abstract

Humans and animals adapt to space flight conditions. However, the adaptive changes of fully formed organisms differ radically from the responses of vertebrate embryos, foetuses, and larvae to space flight. Development is associated with active cell proliferation and the formation of organs and systems. The instability of these processes is well known. Over 20 years has passed since the last systematic experiments on vertebrate reproduction and development in space flight. At the same time, programs are being prepared for the exploration of Mars and the Moon, which justifies further investigations into space flight’s impact on vertebrate development. This review focuses on various aspects of reproduction and early development of vertebrates in space flights. The results of various experiments on fishes, amphibians, reptiles, birds and mammals are described. The experiments in which our team took part and ontogeny of the vertebrate nervous and special sensory systems are considered in more detail. Possible causes of morphological changes are also discussed. Research on evolutionarily and taxonomically different models can advance the understanding of reproduction in microgravity. Reptiles, in particular, geckos, due to their special features, can be a promising object of space developmental biology.

## 1. Introduction

The possibility of reproduction in space is of fundamental importance for space exploration given that people may live on space stations for several years or even generations. For example, a flight to Mars can last for up to 30 months. Despite the fact that spaceflight has been carried out for six decades, little is known about the effect of microgravity on vertebrate development. However, conditions in space are very different from those on earth. For example, humans evolved in the 1 *g* gravitational field on Earth, while the space environment entails exposure to microgravity within spacecraft, zero gravity in deep space, and exposure to hypergravity during launch and re-entry [1]. Under “microgravity”, objects appear to be weightless; i.e., microgravity on low orbits is a state in which the acceleration caused by gravity is balanced by centrifugal acceleration, and the force of gravity decreases slightly (for satellites with an orbital altitude of 252–589 km, by 7.4–16.2%). On the International Space Station, all objects are in a state of microgravity because it moves around the Earth in a ballistic orbit. However, the inhomogeneity of Earth’s gravitational field and other phenomena create quasi-static accelerations that reach values of 10^−6^ g.

Gravity is only one of the factors that influence the formation of complex organisms. Factors such as the Earth’s geomagnetic field, different light cycles during the day and year, and the planet’s background radiation must also be taken into account [2]. The effect of these factors on mammalian reproduction is largely unknown. Space travel poses multiple potential hazards to reproductive health, including exposure to ionising radiation, microgravity (zero gravity), hypergravity, psychological stress, physical stress and circadian rhythm disruption [1]. Space flight-induced developmental pathologies are not clearly defined for humans or other mammals. Findings on the pathology of development in altered gravity are often contradictory [3]. Further, both foetal and maternal factors are involved in the risk associated with pregnancy under altered conditions during space flight.

This review focuses on the effects of space flight in low Earth orbit on vertebrate reproduction and early development. Portions of these data have been published elsewhere, e.g., [3,4,5,6,7,8,9]; however, some new data are included here.

In adult organisms, spaceflight alters some of the normal physiological processes such as homeostatic control of the circulatory system, mineral cycles and tissue metabolism [10]. Exposure to space flight conditions causes numerous physiological effects, including cardiovascular changes, sensorimotor changes (including in vision and vestibular functions), decreased bone mineral density, muscle wasting, changes in lung capacity, increased glomerular filtration rate and decreased sweating, increased percentage body fat, insulin resistance, altered fluid and electrolyte balance [1].

There are many reasons to believe that the developing organism may be more susceptible to the influences of space flights factors than adults. During development, ongoing processes such as active cell proliferation and the formation of organs and systems are sensitive to the influence of various factors. The instability of these processes is well known, even under normal conditions, and numerous pathologies and abnormalities of development are described.

In vertebrates, only a few experiments have been performed in space flights to study fertilisation and embryonic development. A variety of species have been used. Experiments were carried out on fish, amphibians, birds and rodents, which were in space flight at different stages of their development (Table 1) [1,11,12,13,14,15,16,17]. It is important to note that comparing variations in early development over a broad range of species is difficult. Each species has differing rates of development and various sensitivities to environmental conditions [3].

Here we summarise and discuss the effects of space flight conditions on the reproduction and early development of vertebrates. It has been shown that some developmental stages can take place in space. However, at present, no mammalian (or even vertebrate) animal has completed its life cycle from conception to adult in space flight. There are only preliminary results on how the space environment affects the critical phases of reproduction and development: fertilisation, embryogenesis, pregnancy, birth, postnatal maturation and parental care.

## 2. Fishes

Fish have been used in many studies on the effects of space flight, including the influence of microgravity on their development. (Table 1) [15,18]. In early studies, it was shown that the viviparous guppy *Lebistes reticulatus* ceased fertilising its eggs during flight but resumed doing so immediately postflight [18]. Successful reproduction in space was only shown in 1994 on medaka fish (Japanese rice fish, *Oryzias latipes*) during a 15-day mission aboard a space shuttle [13,19]. That study provided the first evidence of mating, fertilization and hatching of a vertebrate in space. In spite of some difficulties with mating in space, live offspring were obtained, which did not show changes in morphology and behavioural reactions [19,20]. Four fishes laid 43 eggs, from which eight fry hatched in space and another 30 fry hatched after landing. Examination of the ovaries of fry hatched in space revealed normal numbers of germ cells, and fries hatched in space produced offspring after re-entry [1]. However, this success was dependent on the selection of fish sent to space [19]. For this experiment, a strain of fish was used which was tolerant to weightlessness. Usually, fish loop when exposed to microgravity. The strain of medaka was found which do not loop at all under microgravity.

Changes in the vestibular system during development under space flight conditions have also been studied (Table 1). In the United States, Skylab 3 experiment and two additional fish studies (on the Apollo–Soyuz Test Project 33 and on the unmanned Soviet biosatellite Cosmos-782), the development of the vestibular apparatus proceeded uneventfully from the late gastrula stage through hatching and early maturity and in young fry, resulting in the normal swimming behaviour of young fry in space flight conditions. However, several young-adult fish that were also used in experiment showed an uncoordinated behavior (abnormal swimming in tight circles, frequent looping sideways). The frequency of the looping declined slowly after the third day of flight. No significant alterations in vestibular morphology were reported by the U.S. investigators [11]. However, marked changes in otoconial membrane morphology were found by Soviet scientists as a result of space flight [21]. More literature on the effects of altered gravity on cranial/neural development in aquatic vertebrates has been reviewed by Rahmann and Slenzka [15].

## 3. Amphibians

Fertilisation and embryonic development can also occur in the space environment for amphibians. However, various abnormalities have been noted in frogs and newts [1].

### 3.1. Anuran Amphibian Species

Anurans have a long history in studies on the effects of space flight and microgravity [11,15]. The advantage of anurans is that their development has been well studied. Also all key stages of ontogenesis can be observed, from the oocyte to tailbud embryos or larvae. In addition, their eggs are relatively large, being more than 1 mm in diameter.

The majority of amphibian spaceflight investigations have been devoted to early embryogenesis, e.g., [11] or vestibular system development, e.g., [22,23]. However, the effects of space flight on the development of other organs and systems are still poorly investigated [24].

In amphibians on Earth, the orientation of their eggs in the gravity field is essential for survival [3,25]. To test this in microgravity, a series of studies were carried out in the 1960s into the early stages of development in American grass (leopard) frog (*Rana pipiens*) on Biosatellite I and Biosatellite 2 and on Gemini VIII and Gemini XlI. For example, during the 11-h Gemini mission, apparently normal cleavage stage *Rana pipiens* embryos were obtained from eggs fertilised on the launching pad [26]. Although it has been suggested that spaceflight conditions disrupt the earliest stages of development such as fertilisation, cleavage, and gastrulation, no clear abnormalities were found in early studies (Table 1). The embryos looked normal upon landing [11,15,26].

However, in all of these experiments, the fertilisation occurred on Earth before launch, so additional research was needed on the period between insemination and first cell cleavage under space flight conditions.

Souza et al. (1994) [27] analysed the fertilisation and development of clawed frogs (*Xenopus laevis*) on board a space shuttle. The ovulation of oocytes was induced by hormone injection in an adult female during the flight, and fertilised eggs were then put into plates in microgravity or in a centrifuge permanently rotating at 1× *g*. Oocytes were fertilised in vitro with spermatozoa previously prepared on Earth. Embryos were fixed for histological study during the flight or kept alive for rearing after landing. The specimen from the microgravity group at the two-cell embryos, gastrulae and neurulae stages, as well as in the swimming tadpole stage, demonstrated normal external anatomy with one exception. Embryos were staged according to Nieuwkoop and Faber (Table 1). Embryos developed in microgravity had thicker blastocoel roofs on the gastrulae stage—an average of 3.7 cell layers in this group and an average of 2.6 cell layers in the group developing under 1 g conditions [28]. Despite that, the neurulae were reported to develop normally. Essentially, all swimming tadpoles from both groups demonstrated normal external anatomy; no differences in developmental rate were noticed between the two groups.

Thus, morphological changes, such as the increased thickness of the blastocoel roof, indicate some changes in cell proliferation during early development in microgravity [1,25]. However, the experiment indicates that gravity is not required for fertilisation and early embryogenesis in this species.

At the same time, in hatchling *X. laevis* larvae raised under microgravity on the US space shuttle, Souza et al. (1994) found significantly larger brain ventricles, head, and eyes [27]. Similarly, Neff et al. (1993) reported enlarged heads and eyes, and an arched back in *X. laevis* embryos raised on a clinostat [4]. The authors believe that the increased head size may have been due to a neural tube or neural plate defect, possibly due to an altered ectoderm contraction wave, and it is likely that the eyes were proportionately larger [3,25].

In addition, astronauts made video recordings of swimming tadpoles during this flight. These recordings and additional recordings made within 4.5 h of landing has shown essentially normal swimming behavior. In particular, there was almost no looping. Postfligt, Souza et al. (1995) revealed the swimming of the tadpoles from the flight group at a lower position in the water column than the 1 g ones [28]. The flight tadpole swimming position was attributed to a difference in lung volume; significantly smaller lungs were revealed in tadpoles from the flight group than in the 1 g tadpoles. Although there were air bubbles in the egg chambers, the tadpoles were apparently unable to find the air–water interface and inflate their lungs.

Our team participated in an 11.5-day experiment on the development of clawed frog tadpoles aboard the Biocosmos satellite [29]. It was devoted to studying the influences of space flight conditions on the brain and sensory system of *X. laevis* larvae. The container with the tadpoles was launched when they were at about stage 25 of development. After landing, all of the animals were at stage 47 of development, which corresponds to young free swimming and feeding larvae [24,29]. The tadpoles had proportionately smaller bodies and longer tails than control larvae. Most of the flight animals had caudal lordosis and consequently swam with backward somersaults. Compared to ground-based controls, their notochords were significantly larger in the cross-sectional area and deformed. Caudal muscle fibres were less dense and degenerated; instead, the cranial muscles associated with buccal pumping demonstrated no alterations in the flight group compared with control samples. The larvae from the flight group demonstrated negative buoyancy at rest after landing, lying on the bottom [24].

**Figure 1:**
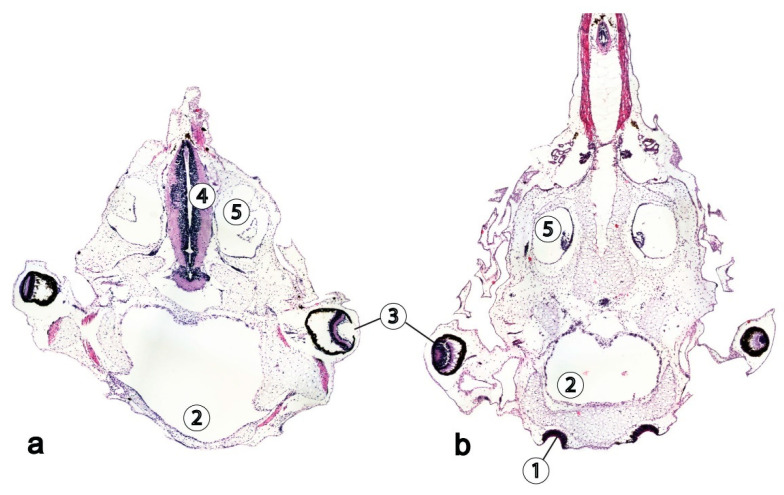
Microphotographs of horizontal sections through the head of Xenopus laevis tadpoles, hematoxilin and eosin. (**a**) Flight group (**b**) Control group. 1—olfactory placode, 2—oral cavity, 3—eye, 4—brain anlage, 5—otic vesicle.

Development of the brain and special sensory systems of *Xenopus laevis* larvae (Figure 1) were also altered under space flight conditions. The total volume of the brain, including the ventricles and grey and white matter was 7% greater in those animals which had undergone the space flight than in the controls [24,29], which coincides with the observations of Souza et al. (1994) [27] and Neff et al. (1993) [4]. However, no changes in the volume of grey matter were shown in the larvae of *Xenopus laevis* post exposure to microgravity. The increased volume of white matter and decreased volume of the retina, olfactory placodes and VIII cranial nerve ganglion size were also revealed. The volume of white matter in the flight group was 30% higher than in the control group. The increase in the volume of white matter seemed to be due to a decrease in the volume of the brain ventricles, which were somewhat smaller in the flight animals than in the controls. The cause of the changes in the grey/white matter ratio remains unclear, but is likely due to the effects of space flight on the morphogenesis of the nervous system. 

In this study, special attention was paid to changes in the size of the cerebral vascular plexus. Previously, the redistribution of cerebrospinal fluid and blood flow was revealed in the animal and human organisms under conditions of weightlessness in favour of the upper part of the body (head) at the expense of the lower limbs and pelvis. However, the volume of the vascular plexus in *X. laevis* differed unreliably, whereas its surface area measured inside the cerebral ventricles was 34% greater in the group exposed to the flight. The increase in the surface of the cerebral vascular plexus may be regarded as an adaptation of the developing brain to weightlessness [29].

At the same time, the volumes of the retina, ganglion of the VIII nerve, and olfactory placodes in weightlessness decreased by 60, 22, and 17%, respectively, compared to the control group. The differences in the volume of the ganglion of the VIII nerve are understandable: since it is associated with the vestibular and otolithic systems of the inner ear, a change in the weightlessness of afferent signals could affect the ganglion’s morphological formation. However, there is no clear understanding of delays in the development of eyes and olfactory placodes.

In addition, the normal asymmetrical development of the cerebral structures of *Xenopus laevis* was unaffected by spaceflight [29]. In amphibians, morphological brain asymmetry first appears at the neurulation stage. This asymmetry was retained during all postembryonic (larval) stages, in both the flight and control groups, in the retina, the ganglion of the VIII nerve and the olfactory placodes. The right-side asymmetry of the investigated structures was 2–9%.

Overall, this experiment revealed that space flight affected tadpole growth and development, both in space and postflight. Some differences with the results of Neff et al. (1993) [4] and Souza et al. (1994, 1995) [27,28] underline, however, the fact that the effects of altered gravity on amphibian organogenesis may differ greatly depending on the developmental stage examined [24].

Special attention was paid on the development of the vestibular sistem. The effects of microgravity on the development of the vestibular apparatus of *Rana temporaria* and *Xenopus laevis* were studied in a series of experiments by Vinnikov et al. [22] who sent into space eggs that had been fertilized on the ground. Only minor changes in the vestibular system, some utricular otolith enlargement, and a tendency for greater asymmetry between the left and right otoliths in some larvae were revealed in the embryos, which did not reach orbit until the blastula stage [22].

Similar results were obtained in *Xenopus laevis* experiment in the Spacelab DI mission in 1984. Postflight, no difference between a flight group exposed to microgravity, a flight group provided with artificial gravity, and a ground-based control group, except for a peculiar swimming behavior in flight group (i.e., the tadpoles swam in small circles was shown) [11].

In the Andromeda 2001 mission (Table 1), some *Xenopus laevis* developed a tail lordosis, like in the Bion-1 experiment (see above). However, significant effects on the shape of otoconia and on the number and size of calbindin-expressing cells of the labyrinthine maculae cells were absent. Therefore, it was postulated that the response of the vestibular system of *Xenopus laevis* to microgravity is linked to processes within the central structures of the vestibular system rather than in peripheral structures [30].

In general, effects observed in *anuran* amphibian species were dependent on the developmental stages at which the animals were launched and on the rearing devices used during the space flights [31].

### 3.2. Urodele Amphibian Species

Similar results were obtained when using urodele species. The newts are a perfect model animal for developmental studies in space experiments, due to the female’s ability to retain live sperm in her cloaca for up to five months. This allows her to be inseminated on Earth, and later (in space) undergo fertilisation, which can be induced through hormonal stimulation [12,32].

#### 3.2.1. Japanese Red-Bellied Newt

Adults and larvae of the Japanese red-bellied newt species (*Cynopus pyrrhogaster*) were used in the first urodele experiment. The so-called AstroNewt experiments were performed on board the Shuttle Columbia (STS-65) on the Second International Microgravity Laboratory (IML-2) in 1994 and on board a Japanese Space Flyer Unit put into orbit in 1995 and retrieved by a Space Shuttle in 1996 [33,34]. The aim of these experiments was to study the development of urodele eggs. Before the space mission, females were inseminated on the ground, one to four months before each of the space experiments, and then maintained at 8 °C until launch. Their eggs developed and matured in the microgravity environment.

Embryos were all described as normal based on video taken in orbit on the sixth, eighth and eleventh flight days. Similar developmental rates in the flight and control groups were revealed using morphologic analysis. Otolith volumes and sensory epithelia areas were measured. The 3D-reconstructions showed the larger mean of the endolymphatic sac and duct volume, and a larger average volume of otoconia in the flight-reared larvae in comparison with the similar developmental stage in ground control ones. The otoconia emerging in the endolymphatic sac were also reported to be developing faster in larvae from the flight-reared group.

#### 3.2.2. Spanish Ribbed Newt

The embryonic development of Spanish ribbed newt (*Pleurodeles waltl*) eggs oviposited in space flight was also studied in experiments on board the MIR station (see Table 1) [12]. The experimental data included video recording, and the results of morphological, histological and immunocytological studies; a subset of newt larvae was kept alive for laboratory rearing upon landing [32]. Embryos and larvae were staged according to the chronological table of development of the *Pleurodeles waltl* according to Gallien and Durocher [31].

The FERTILE missions’ experiments provided evidence of *P. waltl* fertilisation under microgravity conditions without parthenogenesis or gynogenesis [32]. The developmental success was defined as the number of two-cell stage or later stage embryos divided by the total egg number for both of the FERTILE space missions. The developmental success calculated for both space missions was 56% [32]. The time from egg deposition until hatching was similar for the flight and ground control group at 18 °C. The results suggest that conditions on the MIR station were appropriate for newt fertilisation and development.

However, in the FERTILE I, FERTILE II and NEUROGENESIS experiments, structural alterations were observed for cleavage and neurulation [35]. Irregular segmentation and abnormal neural tube closure were also observed [32]. Specifically, the neural tube incompletely closed at the head and trunk levels. Cell adhesion was abnormal during the cleavage and neurulation stages, but did not alter later onthogenesis nor were they lethal [32].

Eighty-one percent of the embryos had this neural tube defect and forty percent of the animals had microcephaly at the early tailbud stage (Stage 31), which may be related to neurectodermal cell loss during neurulation. In a 1× *g* centrifuge on board the shuttles, only 28% of the embryos showed this defect [3,12]. Exposure to microgravity also led to substantial microcephalic phenotypes in some of the experimental embryos, including acephaly (i.e., three embryos) or severe microcephaly. Furthermore, open neural tubes, sometimes with a loss of neurectodermal cells, were presented in the majority of embryos from the flight group at the end of neurulation (stage 20). Interestingly, this effect was specific only to the neural ectoderm [12]. The embryonic epidermis cells, which are differentiated from the ventral ectoderm, demonstrated normal cell adhesion during neurulation in the group exposed to microgravity. Since both the dorsal (neural) and ventral (epidermis) ectoderm originate from the thickened blastocoel roof (see also experiments on *Xenopus laevis* eggs above), the authors speculated that the ventral (epidermis) ectoderm quickly obtained strong epidermal junctions, such as tight junctions and desmosomes, while the neurectoderm had a different developmental pattern. Therefore, the abnormalities of cell adhesion and neural tube closure tissue evidently led to the microcephalic alterations.

The morphogenesis of other embryo organs and systems was reported to be normal under microgravity conditions, including in embryos with acephaly or severe microcephaly, which nevertheless possessed normal trunk and tail development. Outside of the acephalic and microcephalic embryos, further morphological and functional development was not interrupted by the aforementioned abnormalities. A developmental time-course of the majority of normal hatching and swimming larvae from the flight group exposed to microgravity aboard the MIR station, was similar in comparison with the 1× *g* centrifuge control embryos (also on the MIR station) and l *g* ground control ones [12]. Cephalic organogenesis took place correctly for 99% of the embryos that developed in microgravity up to stage 40, with only 1% presenting cephalic abnormalities. The main five subdivisions of the brain were reported morphologically normal, and early ear and eye organogenesis was also described as identical for the flight microgravity and 1 *g* control embryos. This included the normally distributed GFAP-immunoreactive astrocytes within the developing retina.

Collectively, these results indicate stage-specific alterations in embryos exposed to microgravity aboard the MIR station. However, the embryos demonstrate the ability to compensate for nervous system abnormalities [35]. From video taken on board the MIR space station, it was clearly that embryos with such abnormalities developed into hatched larvae [31]. Thus, those abnormalities were temporary and subsequently reversible, and outside several acephalic and microcephalic specimens, ontogeny proceeded quite normally for microgravity-exposed embryos. The live larvae obtained after landing displayed normal morphology and swimming behaviours [31]. During all postflight development up to adulthood on Earth, no differences or abnormalities were found for the specimens exposed to microgravity on the MIR space station.

The progeny of newts from the flight groups were observed up to the second generation. Delayed metamorphosis was reported for specimens from the Cassiopée mission [31], while the percentages of fertilisation and developmental patterns of offspring stayed within normal ranges for the control animals. No genetic or morphological abnormalities were observed in the offspring and the developmental time to metamorphosis was normal to control [31].

In conclusion, amphibians which hatched in space can live and reproduce normally after returning to Earth. These results, based on the urodele *Pleurodeles waltl*, are consistent with the data received earlier from the anuran *Xenopus laevis* and the fish *Oryzias latipes* developmental studies [31] with the majority of embryos able to normally complete differentiation and morphogenesis [28,35,36].

However, the early stages of embryogenesis until the early tailbud stage are sensitive to an absence of gravity. These results, documenting abnormalities that appear during the early ontogenic stages of *Xenopus* [4,24,27,28,29] and medaka [19]. The delay in development in space flight was observed in the experiments with amphibian larvae. At the same time, in early development, the plasticity of the biological system allows to compensate alterations and provide the high persistence of the organism against many disruptions and risk factors [25].

## 4. Reptiles

**Figure 2:**
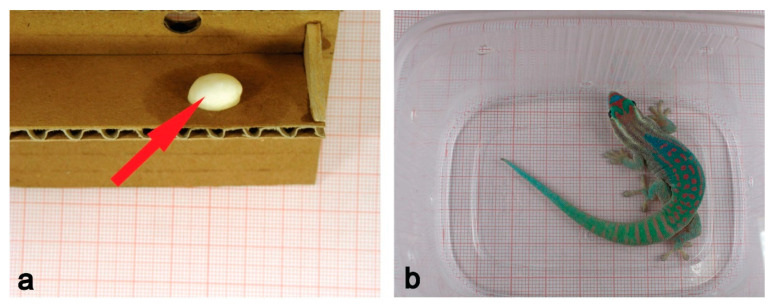
(**a**) Unfertilized egg of thick-toed gecko laid by a female on the wall of a flight container (Foton-M3, 12 days flight, 2007). (**b**) Male ornate day gecko before the flight experiment onboard unmanned spacecraft Foton-M4 (44.5 day flight, 2014).

Reptiles are one of the least studied vertebrates in space biology. In 2005, a successful 16-day orbital experiment of five thick-toed geckos (*Chondrodactylus turneri*, including four females and one male) was carried out on-board the unmanned satellite Foton-M2 [37]. After landing, unfertilised eggs were found on the inner lining of the container with geckos. Unfertilised eggs (Figure 2a) or traces of them after being eaten by geckos were also found on the walls of containers after orbital experiments with thick-toed geckos on-board Foton-M3 (12 days, 2007) and Bion-M1 (30 days, 2010) in which only females participated. Thick-toed geckos compare favourably with many other species of geckos due to both unpretentiousness and ability to attach to surfaces, which they retain in weightlessness. In addition, in normal Earth conditions, thick-toed geckos bury their eggs in the sandy substrate, but we revealed, that without such substrate, thick-toed geckos stick eggs to surfaces both in weightlessness and in ground control group, and this happens quickly and firmly (previously unpublished data). This simplifies their reproduction in microgravity. Perhaps one of additional advantages of geckos is that they are arboreal, since until now, mainly aquatic and terrestrial vertebrates have been used for space research.

Unfortunately, thick-toed geckos feed exclusively on live insects, and it was technically difficult to provide them with food of the right quantity and quality for more than a 30-day flight. Therefore, ornate day geckos (*Phelsuma ornata*, four females and one male) were chosen as a preferred taxa for a 60-day experiment to study gecko reproduction in space flight (Figure 2b).

Like thick-toed geckos, ornate day geckos adhere their eggs to surfaces; however, unlike thick-toed geckos, they are able to eat pasty food of a fruit-grain basis for a long time without harm to their health.

**Figure 3:**
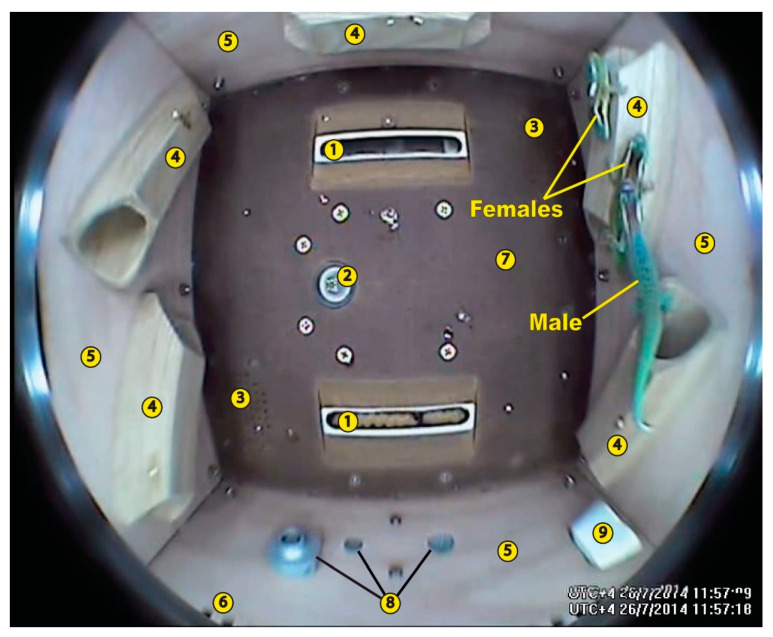
Video capture of ornate day geckos, sexual behavior in weightlessness: mounting of a male on a female, seventh day of the flight (Foton-M4, 2014). 1—Feed box; 2—water bowl; 3—heating zones; 4—tubular shelters for geckos made of American oak; 5—research and support block (RSB) walls lined with American oak; 6—vents for ventilation and waste collection; 7—RSB floor made of textile laminate (a fabric reinforced laminate); 8—O_2_/CO_2_, temperature and humidity sensors; 9—cuttlefish shell as an additional source of calcium. Video camera and a fan are located on the ceiling of the container out of sight.

The only attempt to reproduce ornate day geckos in microgravity was an experiment on-board the Foton-M4 satellite, which received the name “Sex-Explorer” in the mass media. It was launched on 19 July 2014 and remained in space for only 44.5 days instead of the planned 60. Since ornate day geckos are more demanding with regard to moisture and water availability, their container was equipped with not only a feeder, but also a drinker, as well as a source of ultraviolet light that is necessary for the optimal condition of the animals. Nevertheless, all geckos died before landing. Unfertilised eggs were only found in a ground-based control experiment. The cause of death could be either low temperature, or a gradual drop in the partial pressure of O_2_ and an increase of CO_2_ concentration. However, there were still elements of sexual behaviour seen in flight videos (Figure 3) which indicate that under more favourable flight conditions, lizard reproduction in space might be possible [38]. *Phelsuma ornata* remains the second vertebrate species after medaca fish (*Oryzias latipes)* and the first amniote species in which sexual behaviour in weightlessness was recorded.

**Figure 4:**
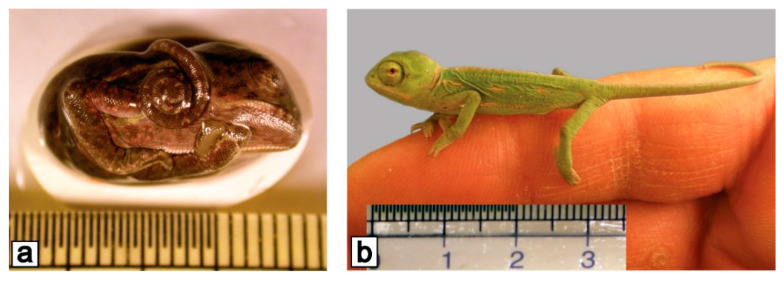
Veiled chameleon: late embryo before hatching (**a**), newborn cub (**b**). The photographs were taken in preparation for an unrealized experiment on the development of chameleons in space flight (own data). The scale bar is the same in both images, the smallest division is 0.5 mm.

We have subsequently started preparing an orbital experiment for the development of veiled chameleon (*Chamaeleo calyptratus*) embryos (Figure 4) in space (previously unpublished data). The scientific equipment (BIOCONT-FEG) is supposed to provide incubation for nine units of biomaterial for 60 days at +27 ± 2 °C and humidity of 80–100% in a substrate consisting of vermiculite with a pressure grid that excludes flotation and displacement. It is assumed that the equipment would also provide the necessary gas exchange through continuous ventilation and maintain the environmental parameters, not only by heating, but also by cooling the incubator in the case of emergency situations using Peltier elements.

The advantage of geckos and chameleons is the autonomy of embryo development from the mother’s body, which can be an additional source of artefacts under stressful conditions. The relatively low level of metabolism and the long ontogenesis of the selected species make it possible to adapt chameleons to long-term microgravity exposure and assess its influence on the normal development of these vertebrates. An additional advantage of chameleons for embryonic experiments in space is the sensitivity of embryos to gravity, which makes it possible to assess the influence of gravity on the early ontogenesis of an amniote. Still now, the project remains unrealised for financial and organisational reasons, but we hope to fulfil it in future.

## 5. Birds

Japanese quail (*Coturnix japonica*) eggs have orbited the Earth in several experiments [39]. Investigations of bird embryonic development in orbital flight experiments is important for a general understanding of the biological role of gravity in species biology and evolution [40].

The first developmental experiment with quail eggs took place in 1979 on board the Soviet satellite Cosmos-1129. Quail eggs were incubated under orbital flight conditions for 12 days, which is two-thirds of the species’ embryonic period. However, most eggs were broken during landing [41]. The most important result of the study was the proof of principal regarding the possibility of the successful incubation of quail eggs in microgravity. Two interesting facts were revealed: the death rate of embryos was greatest in the early stages of development, and there was a lag in the timing of the development of embryos in the flight group.

In March 1990, quail eggs on the MIR space station were successfully incubated and hatched [40]. Later, a series of experiments with the quail eggs was carried out. These experiments on-board the MIR station showed that normal quail embryogenesis is possible and viable progeny can be obtained, although there is a considerably lower hatching rate and a higher frequency of anomalies. The embryos had developmental abnormalities in their eyes, brain and beak, leading to microphthalmy, anophthalmy, and other violations of normal eye morphogenesis, consisting of changes in the proportions of the pigment epithelium and retina [42]. Anencephaly, displacement or crossing of the beak plates was also observed. The sensitive period for eye development in quail is the second to fourth day of incubation. Also, it is known that eye development is easily impaired by unfavourable conditions. Therefore, eye anomalies were considered an indicator of general unfavourable conditions for embryo development.

The embryos and hatchlings had no significant deviations of the morphological indices of body size and weight [43]. Nevertheless, indirect effects of weightlessness on final results of incubation cannot be excluded. A considerably lower hatching rate and a higher frequency of anomalies were probably connected with the indirect influence of weightlessness on the embryo development (for example, through the impairment of the water-salt balance and the redistribution of fluid in the body of the embryo) [40,42]. Moreover, the results of histological studies have shown that Japanese quail embryos in space flight have a lag in the development of the spinal cord. These are expressed in a delay in morphogenesis, in particular, in the presence of incomplete proliferative activity of migration processes. However, the hatching of chicks occurred on the seventeenth day, just as on Earth [44]. Quail raised in microgravity are unable to peck normally [45], and this behavioural deficit may be related to vestibular malfunctions [46]. Other disorders in quail development include: muscle distrophy and atrophic changes [47], delay in the development of the thyroid gland [48], abnormal liver histogenesis in the early stages [44], lag in osteogenesis [49,50], weak development of connective tissue in the gastrointestinal tract stroma, and focal hyperplasia of the duodenal epithelium with a predominance of proliferative processes over differentiation [25,51].

Calcium intake from the shell of a developing quail embryo in microgravity was reduced during the early stages of development (day 4). Calcium utilisation by developing embryos incubated in microgravity was impaired by 12.6% compared to embryos incubated on earth in a controlled laboratory environment. These many impairments in calcium balance were believed to be due to unidentified factors of the microgravity environment [16,52]. Thus, some of the observed alterations may be associated with disturbances in calcium metabolism, which were observed in space flights [25]. However, by the end of embryogenesis (days 14–16), there were no essential differences in skeleton development excluding delay in ossification between the control and flight groups [50].

## 6. Mammals

It is difficult to use mammalian embryos to study the direct effect of weightlessness on development, due to the intrauterine development. The influence of the mother’s body is difficult to separate from the direct effects of weightlessness on the foetus. Because of the complexity of life support for mammals, research into their reproduction in space has not progressed as far as in other animals. There have mainly been experiments on rats, with the course of pregnancy in mice studied in only one flight. Pregnant mice have been used only once thus far, on the Neurolab mission [17]. Prenatal and postnatal alterations and even transgenerational effects, which could be limit their survival, were hypothesised as a potential consequence of microgravity exposure during early development. In fact, no microgravity simulation effects on mice fertilisation were reported in two studies, and the negative effects on early development after microgravity simulation were provided in four reports, with results varied according to experimental microgravity conditions [53].

### 6.1. Fertilisation and Preimplantation

The first experiments on the prenatal development of mammals under space flight conditions were carried out with rats on board of an unmanned Cosmos-1129 biosatellite. Serova and Denisova (1982) [54] reported the results of an 18.5-day space flight in which five female and two male rats were flown. The males and females were initially separated, but the separator was removed during the mission, allowing the rats to mate; however, their interaction was not monitored. After landing, it was reported that two of the five female rats showed signs of early pregnancy. Although laparotomy performed on the females showed that ovulation had taken place, there was no clear evidence of embryo resorption. Factors other than the spaceflight condition itself were proposed to have caused the experimental outcome based on the fact that there were also no pregnancies in the ground controls [55]. After flight, the rats from the flight group were mated with nonflight partners; normal litters were obtained in all cases. The data showed that the ovulation, copulation and fertilisation processes could occur under the space conditions, although some processes deviated from what normally occurs on Earth.

The fertilisation of mice in space was studied in the Neurolab mission. The experimental mice were reported to look healthy in flight, but pregnancy did not occur and pre-implantation embryos were aborted. Unfortunately, no postflight information for these mice and offspring was provided [53]. At the same time, in simulated microgravity conditions using a horizontal clinostat device, successful fertilisations were reported and healthy litters were born [6].

In addition, the radiation level on the ISS remains about a hundred times higher than on Earth. Radiation damage to chromosomes in spermatozoa can lead to a variety of problems, from infertility in men to an increased likelihood of cancer in the offspring.

The male reproductive system was reported to be affected for the rodents by the space flight conditions. Seminiferous tubule degeneration and decreased epididymal sperm counts were described for the mice during space flight and in rats under experimental microgravity. Furthermore, significantly decreased serum and testicular concentrations of testosterone were reported for male Wistar rats within 8–11 h of the 14-day flight on-board COSMOS 2044. The number of spermatogonia of the fourth stage in seminiferous tubule cross-sections was significantly decreased by 5–10% in the rat testes from the flight group compared to vivarium and simulated launch control in the COSMOS 2044 and COSMOS 1887 experiments [1].

Wakayama and colleagues also experimented with mouse sperm that had been frozen to −95 °C for more than nine months on the ISS. The DNA in the “space” spermatozoa was severely damaged, but after the procedure of artificial insemination and egg transplantation into surrogate mothers, several dozen mice were born, which were quite healthy and who themselves were later able to produce healthy cubs. On the other hand, the ISS is still within the boundaries of the geomagnetic shield that protects the Earth from cosmic radiation. If someone needs to go into deeper space—for example, in flying from Earth to Mars, then the genetic defects in the germ cells may be substantial [56].

In contrast to the abovementioned data, no alterations in the male reproductive system and sexual behaviour were reported for rats exposed to microgravity during three weeks in space. Moreover, the reproductive function of the rats exposed to microgravity remained unchanged, the offspring of male rats from the flight group were observed to be normal [8].

Alterations in ovarian folliculogenesis, including the disruption of oestrous cycling and impaired in vitro follicle development, were reported in a few space flight and simulated microgravity studies [1].

Analyses of spaceflight effects on the early events of pregnancy remain an important area for future research [17].

### 6.2. Maternal Influences on Development in Mammals

For the three spaceflights that have carried pregnant rats into space, the rat pregnancies were relatively well-established prior to launch [17].

#### 6.2.1. The Cosmos-1514 Biosatellite

In 1983, mammals were carried on the Cosmos-1514 biosatellite, which, for the first time, solved the fundamental question of the possibility of mammalian foetal development in microgravity [18]. The animals spent 13–18 days of pregnancy in space, which is about one quarter of the rat’s intrauterine development. This is the time of active growth of the foetus, during which the formation of its nervous and endocrine systems, skeleton, muscles, and internal organs occurs. Five female rats of the flight group were dissected; the remaining five females were allowed to continue their pregnancy to term. When the rats were dissected on day 18 of pregnancy, the flight group did not differ significantly from the controls in preimplantation or total embryo deaths. No dead fetuses were found in any of the groups; however, the flight and synchronous control females produced more fetuses and placentas showed hemorrhages caused evidently by factors associated with reentry.

Of the five flight females that were allowed to give birth, four delivered live litters and one delivered a stillborn litter [18]. In this flight rat, the birth process lasted two days, due to general asthenization, muscle weakness, and the presence of one very large fetus (greater than 7 g). Since the mother was unable to deliver (it was first in the birth canal) for a long period, the other fetuses—full-term and normally developed—died from anoxia. The largest neonate rat (first in the birth canal) was found to have hydrocephalus. Litters from flight mothers showed increased mortality during the first postnatal week.

Male and female pups that survived to adulthood reached puberty and gave birth to offspring [1]. However, new-born rat pups showed a lag in body weight gain and skeletal ossification compared to the ground controls, which, however, was compensated for on the first day of readaptation to Earth’s gravity. The flight neonates did not display hemorrhages observed earlier in the fetuses. This suggests that the hemorrhages were slight, and the tissue changes they caused, reversible [18].

The general sensory function of the rat pups aged from the postnatal day 1 to day 20 was examined post flight [57]. No significant changes were described for tactile and olfactory sensitivity, vestibular function, auditory responses or visual sensitivity. The delayed development of responsiveness to 40 kHz tones and increased vestibular sensitivity to angular acceleration in a rotation test were the only alterations reported for the pups from the flight group. The significance of these observations is not clear [17,57,58].

At the same time, macroscopic examination of the rat foetal brains from the flight group revealed no alterations in the brain morphology between the flight and control specimens [59]. Interestingly, in the rat brain, differentiation into the main neuron type is completed at E18 (E—day of embryonal development), the developmental process at this stage is characterised by active cell migration and, correspondingly, layer differentiation and nuclei formation, neurite outgrowth and sprouting, the main tract formation, and vessel germinating. However, postflight analyses of foetal tissue revealed mitotic figures in cerebral cortex of the brains of flight foetuses, but not in synchronous or vivarium controls. This was interpreted to indicate either an aberrant or decelerated schedule of neuronal births and signs of retarded cellular development and migration [55].

The cortical plate average thickness of the medial, dorsal, and lateral parts of the wall of the rat pallium was reported to increase in the flight group, which was explained by the altered rate of cell migration. Foci of degeneration of the cell layers were not found [59]. Quantification of capillaries in the striatum of fetuses of the flight group revealed a 40 and 59% increase in their number in a unit area, compared to the vivarium and ground-based synchronous control groups respectively. An increase in the number of capillaries in the fetal striatum, evidently compensatory in nature, was another sign of some deficiency in their brain oxygenation [18].

There were also changes in ocular morphology and vestibular nuclei anomalies. The blood vessels and capillaries of the foetal brains were reported to be smaller and thinner in the flight group based on alkaline phosphatase data [59].

In general, the results of the embryological experiment on the Cosmos-1514 biosatellite and the accompanying ground-based experiments revealed both the possibility of normal development of the mammalian foetus under the influence of weightlessness, and the possibility of serious changes in individual foetuses. Most of the animals endured flight conditions without irreversible pathological changes and complications during re-adaptation to 1 *g*, but serious changes occurred in some individuals, such as the birth of a dead litter or weakened rat pups that died in the first days of life [60].

#### 6.2.2. NIH.Rodent 1 and NIH.R2 Experiments

Pregnant mammals were also flown in space on the space shuttles during the NIH.Rodent 1 and NIH.R2 missions (Table 1). In both missions, 10 pregnant rat females were launched at midpregnancy (ninth gestational day for NIH.R1 and 11th—for NIH.R2) and landed close to the term (E20) of the rats’ 22-day pregnancy [17]. Daily video recordings on-board the space shuttle were performed by the crew [58]. No significant alterations in the functional statuses of the rat dams and of the course of the pregnancy. The pregnancy weight gain, duration of pregnancy and parturition, ovarian and pituitary weights, numbers of healthy or atretic ovarian antral follicles, number of corpora lutea, serum concentrations of progesterone or luteinising hormone, follicle-stimulating hormone, and litter sizes were all comparable to the control groups [1]. In the NIH.R2 experiment, pregnancy duration and litter size were also not changed by space flight [1].

Our team took part in the study of new-born rat pups that had passed half of the prenatal period under conditions of weightlessness on the maternal organism in the NIH-R1 experiment and were born on Earth two days after completion of the space flight [61]. As rats typically give birth on day 22 of gestation, the developing rats were subjected to space flight conditions for the majority of their embryonic and foetal life. These times correspond to developmental stages during which neurulation and organogenesis take place.

Increasing the exposure of pregnant females to 11 days of space flight did not lead to visible anatomical abnormalities in foetal development [25,62]. The weight of litters from the flight group was similar compared with control groups [1].

**Figure 5:**
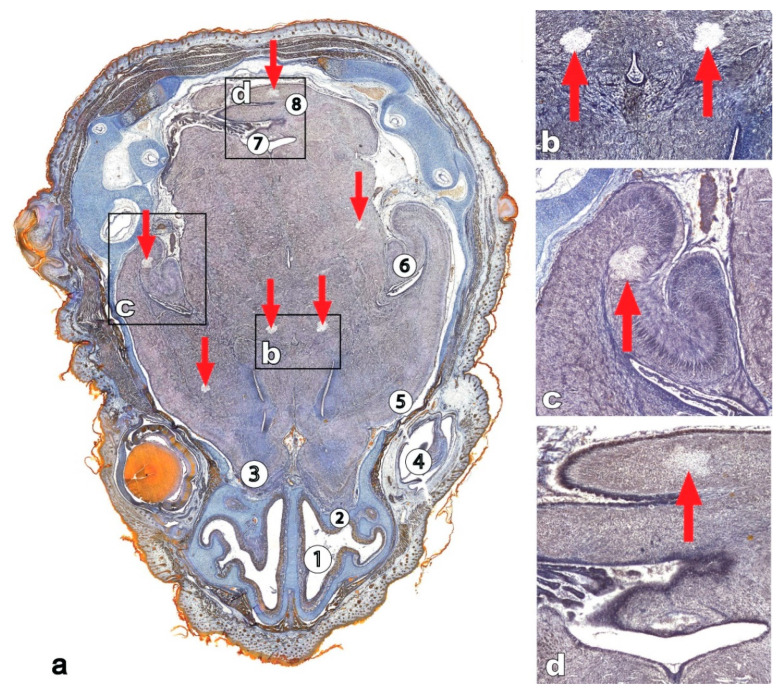
Horizontal section through the head of *Rattus norvegicus* newborn pups (flight group (NIHR1). Mallory staining. Foci of degeneration (red arrows) are shown at low (**a**) and a higher (**b**–**d**) magnification; the rectangular on A highlights the region shown on (**b**–**d**). 1—Nasal cavity, 2—olfactory nerve, 3—olfactory bulb, 4—eye, 5—cortex telencephali, 6—hippocampus, 7—fourth ventricle, 8—cerebellum.

In the rat pups from the flight group, proliferative-migration abnormalities of brain development and neuron differentiation were revealed using histological and morphometric analysis. The histological analysis of pups from the flight group revealed neuronal degeneration with cell loss in all of the brain regions studied (e.g., telencephalon, see Figure 5) in 70% of the pups. The foci of altered nervous tissue with a decrease in the neuron number and signs of nerve cell destruction (i.e., appearance of apoptoid bodies and cellular detritus, accumulation of glial cells around collapsing neurons) were shown in various divisions of brain and spinal cord. The areas of neurodegeneration were about 50 to 400 μm in size. Such areas were often seen next to the zones of otherwise normally developed neural tissue. The lack of neuroglial proliferation in these areas of neurodegeneration suggests that the pathology may have developed in very early embryos. Separation of the white matter was also shown. Since such alterations have not been detected in other experiments, it is possible that they are due to some unique features of this NIH-sponsored flight.

In the brains of rat pups in the NIH-R1 experiment, special attention was paid to five structures: superior (SQC) and inferior quadrigeminal colliculi (IQC), medial (MHN) and lateral habenular (LHN) nuclei and caudate nucleus (CN). These structures were chosen because they lie in different brain divisions (IQC and SQ—in mesencephalon, LHN and MHN—in diencephalon, CN—in telencephalon). This allowed the experimental influence of the space flight on major brain divisions to be estimated. The nuclei are also included in different functional complexes of the brain: the SQC are part of the visual system, IQC are part of the auditory system, while LHN and MHN display extensive liaisons with the limbic system and interact with olfaction and epiphysis of brain. The CN has functional links with the limbic system and is the target of the nigrostriatal system. In this part of the review, we will focus on features of sensory and motor system development in the NIH-R1and NIH-R2 rat foetuses after the influence of space flight.

1. Vestibular system: Vestibular stimuli proceed separately from the inner ear. The otoconia of macule and saccule percept linear acceleration, which correspond to the gravitational field perception. The semicircular canals process angular acceleration afferentation providing the perception of head rotation. The microgravity exposure seems to affect these two vestibular system subdivisions differently. Alterations in linear acceleration perception are expected after a long-term space flight in adult mammals. However, deprivation of the gravistatic input to the otoconia, and increasing of the sensitivity to angular acceleration were hypothesised for the intrauterine development in microgravity, because of rotations of the dam’s body in space flight conditions [58].

In the NIH.R1 experiment, the embryos were exposed to weightlessness from gestation day 9 (before vestibular ganglion neurons contact vestibular nuclei) to gestation day 19 (near the time when the vestibular system becomes somewhat functional) [46]. In specimens from the flight group, the utricular and saccular axons were large unbranched and in growth cones ended in contrast to wide sprouting axons in the control group. Furthermore, the facial sensory neurons in foetuses from the flight group demonstrated wide branching fibres going to the utricle, which were absent from the control specimens [46,63]. Microgravity was speculated to affect gravistatic sensory neurons through developmental delay, leading to a decrease in mature afferent synapses in foetuses from the flight group [58]. However, the total synapse quantity of the medial vestibular nucleus remained unchanged in the flight specimens compared to the synchronous controls. Other vestibular afferents i.e., for angular acceleration perception were suggested to compensate for decreasing gravistatic inputs by increasing of synaptic number from the semicircular canals. Nevertheless, a postflight phenotype was postulated to characterise the reduced gravistatic sensitivity with a corresponding reduction of the saccular inputs and increased sensitivity to angular acceleration with increased semicircular canal afferents [58]. Interestingly, in the Cosmos 1514 experiment, anomalies of vestibular nuclei were reported in the pups of the flight group (see above [59]).

In the NIHR1 and R2 spaceflight mission, vestibular-mediated behavioural responses were investigated in the pups aged from the first to the fifth postnatal days [58]. The contact-righting reflex, which designed stereotyped rotation movements turning the body over supine to prone on a solid surface, was similar in flight and control pups. However, water immersion righting tests revealed less frequent righting in neonates from the flight group. These results seem to be due to the removal of tactile and proprioceptive inputs in water, which could compensate for vestibular impairment in the case of solid surfaces. This behavioural deficit was also short-lived and the complete response recovery was demonstrated by neonates from the flight group on P5 [17]. Moreover, Serova, [10], found a decrease in tactile and vestibular sensitivity in the new-born spaceflight-exposed subjects. In P5 (postnatal day) pups, however, all differences between the experimental and control animals disappeared, suggesting postexperimental recovery.

Collectively, these findings provide evidence for the selective disruption of vestibular-mediated responses after prenatal exposure to spaceflight [17].

Together, research provides preliminary evidence that gravity and angular accelerations, produced by the mother’s movements, may shape prenatal organisation and function within the developing mammalian vestibular system [58].

2. Auditory system: Inverted asymmetry of the IQC was revealed in P0 pups from the flight group. The volume of IQC was significantly larger on the left side in P0 pups from the control groups, whereas the right-side IQC was larger in pups developed under spaceflight conditions and in control E20 foetuses. It is unclear if these changes are caused by an influence of weightlessness, or from nonspecific effects related to the conditions of spaceflight [61].

3. Visual systems: The superior quadrigeminal colliculi (SQC) are part of the visual system. In mammals, the visual images are projected through the lateral geniculate body into the cortex. The fibres of the optical nerve end inside the lateral geniculate body, and their collaterals are directed into the optical layer of the anterior colliculi of the quadrigemina. In many mammals, the SQC develops asymmetrically with dominance of the left anterior colliculi. Our results show that the left anterior colliculi were also larger than the right ones in control pups. However, in the flight group, subjected to microgravity, the asymmetry of the anterior colliculi was reversed. The volume of the colliculi on the right side in this group was consistently larger than on the left side. In E20 control embryos, the right-side pattern of the SQC was also observed [61].

The retina of experimental animals in the spaceflight group was thinner, probably as a consequence of a thinning out of bipolar retinal cells [64]. However, whether such changes can significantly affect vision during long flights in foetal or adult subjects is not known. It is important to note that in other experiments on effects of space flight conditions on rat foetuses, some changes in the visual system were also revealed: in Cosmos 1514, ocular morphology was altered [59].

The superchiasmatic nucleus (SCN) is the brain area involved in circadian rhythms and the study of the in the NIHR2 offsprings exposed to space flight condition during prenatal ontohgenesis evidenced for the developmental delay. Histological examination of the retina revealed no differences in development between the flight and control animal retina at E20, P1, P3, and P8. The pattern of cFos activation within the SCN of the E20 animals indicated that the flight group was significantly delayed from the control group at this age. These differences disappeared by P1 [65].

4. Olfactory system: The habenular nuclei is evolutionarily connected to the parietal eye. In mammalian species with a reduced pineal organ, the habenular nuclei are involved into the olfactory system, having symmetrical anatomy. In control pups, the left MHN was larger. Conversely, in E20 control foetuses and flight pups, the right MHN was larger. The total volume of the medial habenular nuclei was 21% smaller in flight pups, which developed while their mothers were subjected to space flight conditions. An explanation for this, much like in the case of auditory and visual systems, may be an underdeveloped status of the left nucleus in the flight group [61].

Effects of prenatal spaceflight on somatic motor development have not been reported [17]. We were also not able to find any changes in caudate-putamen complex involved in the regulation of movement of the flight pups.

In addition, space flight conditions may delay maturation of the choroid plexus of rats flown during prenatal development. The choroid plexuses secretes 70–90% of the cerebrospinal fluid and is involved in brain homeostasis. Epithelial cells of the choroid plexuses of the lateral, third and fourth ventricles were studied in the NIHR1 foetuses from the flight recovery and control groups. Immunohistochemistry revealed altered distribution of the cytoskeletal protein ezrin, which is responsible for apical cell differentiation, and carbonic anhydrase II, which is involved in cerebrospinal fluid production. The changes observed in flight foetuses were characteristic of delayed maturation of the choroid plexus. Moreover, modified cerebrospinal fluid production was demonstrated for rats exposed to microgravity for two weeks in the space flight experiment [66].

In conclusion, in Cosmos 1513, NIHR1, and NIHR2, there were no serious long term changes in the development or behaviour of the prenatally-flown offspring [17]. However, histological and morphometric analysis of the brain and special sensory systems revealed a delay in brain development in the space flight conditions. These changes are seen particularly in major sensory systems, including the visual, acoustic, vestibular, and olfactory systems. Moreover, some findings clearly indicate that microgravity, and possibly other nonspecific effects of spaceflight, can alter the normal development of the brain itself [61].

#### 6.2.3. Effects of Postnatal Spaceflight

In the NIHR3 and Neurolab experiments, the effects of the spaceflight condition on the early postnatal litters were investigated. However, these experiments became complicated due to poor offspring survival and health problems [17]. The NIH.R3 (1996) experiment flown on STS-72 for nine days was the first flight of developing postnatal mammals. Six lactating dams and their nursing pups were used. Two litters per age group of five, eight or 14 postnatal days were launched, of which only 10% of the five-day postnatal group pups survived, 90% of the eight-day postnatal group and 100% of the 14-day postnatal group. The eight-day pups averaged approximately 25% less body weight than the synchronous control, and the weight of the pups from the 14-day group was comparable to control specimens.

The Neurolab SpaceLab Mission 16-day spaceflight experiment (1998) was focused on the developing nervous system [67]. Rat litters at either eight or 15 postnatal days were used [17]. Many of the experimental pups were abandoned or destroyed by the dams [17,67,68]. More than half of the pups from the eight-day postnatal group died or were moribund and were therefore euthanised during the flight; survivors showed dramatically reduced body weights at landing, whereas the pups from the 16-day postnatal group survived and demonstrated good health [67]. The younger litters demonstrated malnutrition, dehydration and hypothermia during flight, which provides evidence for disruption of the maternal–offspring system; older pups did not show such troubles. The unhealthy pups were eliminated from the experiment and only healthy survivors were taken further in the Neurolab studies. The small sample size weakened the power of the study [17].

Nevertheless, additional results were obtained. For example, two main effects in sensory system on the rats subjected to weightlessness were observed in the eight postnatal days group. Vestibular neuron growth was decreased and cerebellar endings in the vestibular nuclei were underdeveloped. Such effects may have occurred because these connections developed during the time period in which the rats where experiencing weightlessness. The second type of effect was nonspecific and concerned the abnormal distribution of synaptic proteins (synaptophysin and SNAP-25) in the vestibular nuclei of the flight rats. The findings suggest that gravity may be important for the normal development of brain pathways related to balance and posture [69].

Intriguing results were found concerning organisation of the hindlimb representation in the somatosensory cortex of Neurolab pups from the 15-postnatal-day group [70]. The synaptic cross-sectional lengths of layers II/III and Va were larger in the flight group; here, a significantly lower synaptic density was registered in the layers II/III, IV and Va, with a 15.6% decrease in the layers II/III, which was the greatest difference. The four-month readaptation period to 1 *g* revealed the transient and reversible character of these alterations. Some of the changes disappeared, as other novel differences appeared. The significant decrease in the synaptic density of layers II/III and Va was no longer observed after readaptation, whereas the synaptic density of layer IV increased by 13.4% in the flight group. All of these laminar-specific alterations regarded the asymmetrical excitatory synapses of the neocortex. The data suggest a role of gravity in the normal cortical synaptogenesis [17].

Contradictory data was received in the eight-postnatal-day group of Neurolab flight rats in the dendritic branching of the spinal motor neurons examination [71]. Medial motor neurons of the rats from the flight group (P25) demonstrated decreased dendritic branching in comparison to the control. The dendritic reduction was specific to the medial neuron population and was not observed for the lateral motor neurons [17].

Swimming behaviour of young rats was also studied [72]. These findings clearly show that an altered gravity influences the postnatal development of motor function. The nature of the differences between animals reared in space for 16 days and those remaining on the ground reflects an adaptation of the flight animals to the microgravity environment [72]. Moreover, P14 rats flown aboard a 16-day mission showed immature tactics in surface righting until the last day of behavior testing 123 days later [73]. Absence of contextual motor experience in microgravity arrested the maturation of the motor tactics for surface righting. Interestingly, such effects were transient in animals spending nine days (from P15 to P24) in microgravity. These findings suggest that a critical period of development exists where the elimination of gravitational input to the vestibular system can impede the normal development of some reflexes [68] and provide evidence that neurons subserving motor function undergo activity-dependent maturation in early postnatal life in a manner analogous to sensory systems [71].

A complicated study of the developing mouse brain was also conducted in the Neurolab-mission. Foetal mouse brains were labelled during the spaceflight with two markers of DNA synthesis -, tritiated thymidine and bromodeoxyuridine, then fixed and stored in the cold during the rest of the flight. Significant differences were observed in the ventricular zone of the prenatal brain of the foetal mice from the flight group. An increased number of labeled cells was found in the ventricular surface in the mice from the flight group. The 3H-TdR/BUdR ratio was also altered [74,75]. Although the experiment demonstrated that complex studies on brain development could be performed during spaceflight, unfortunately, no more data have been published from the study.

In conclusion, currently, neither mice nor rats have been reared completely in weightlessness. The main difficulty is separating the effect of space flight factors directly on the developing organism from the effects of these factors on the mother. For example, maternal behaviour and the physical relationship between the mother and pups (parental behaviour in space has been studied only in rats) may also change under these conditions. Mother-offspring bonds if disrupted detrimentally affect infant growth and development. Poor maternal care can lead to neural, physiological and behavioural changes, including starvation, dehydration, hypothermia, growth failure, arrested neural development, impaired cognitive function and emotional disorders in her offspring [17]. Maternal behaviour could be altered in spaceflight conditions (see [76]). For example, nursing could be complicated and even broken off, due to mothers and their pups floating free of each other in microgravity. Moreover, sensory deprivation delay sensory system development during space flights.

Data on mammalian ontogenesis, both prenatal and postnatal, in the space flight, are few and based on brief periods of the mammalian life. As such our knowledge on the reproductive function and early prenatal and postnatal mammalian ontogenesis in space is limited. For instance, no births of mammals have been registered in space, so even successful fertilisation and placentation in space flights remains questionable [3,7,17].

Longer experiments in spaceflight conditions will be necessary to determine how microgravity affects the reproduction and development of mammals.

## 7. Human

The results of model experiments indicate that some phases of reproduction and the early development of vertebrates can occur during space flight. The space flight condition possesses many risk factors for human reproductive health. The potential effects of microgravity, hypergravity and space radiation on the male and female reproductive system, hypothalamic–pituitary regulation system, fertilisation and prenatal development are still obscure [1].

To date, pregnancy is a disqualifying factor for space travel due to fears of exposure to radiation and toxins, decompression sickness, the potential adverse effects of weightlessness on early embryogenesis, and the risk of accidents during pregnancy, such as spontaneous abortion, ectopic pregnancy, or premature birth. Therefore, every female crew member is tested for pregnancy during preflight medical examinations. Moreover, female astronauts use oral contraceptives containing oestrogen and progestin to suppress their menstrual cycles. Females may elect to delay childbearing for serial space flights, as the mean age at first space flight is 38 years for females [77]. Thus, the use of assisted reproductive technology is common for female astronauts as a result of advanced maternal age. Only one paper by Jennings and Baker [78] reported data on the reproductive success of female astronauts after space flights and whether it causes any differences in success rates when using assisted reproductive technology in female astronauts and older women not exposed to space flight conditions [1].

NASA’s policy also forbids sex in space, and there have been no confirmed instances of it happening [79].

## 8. Conclusions

More than 35 years ago, a promising program for studying the growth and development of mammals in space flight was published, which has still not lost its relevance [60]. It included:

1. Assessing the impact of weightlessness and hypergravity on various stages of pre-and postnatal development;
2. Assessing the possibility of existence in space flight conditions during the full cycle of individual development and in a consecutive series of generations;
3. Studying the rate of aging, age-related changes in general resistance and reproductive capacity of animals during space flights and after returning to Earth.

Research on evolutionarily and taxonomically different model taxa can significantly advance the understanding of the problems and mechanisms of reproduction in microgravity, bringing its successful implementation closer. These models demonstrate a wide range of advantages and disadvantages. Fish are a well-investigated models for space exploration, but they experience serious orientation problems [80] that affect their behaviour. In addition, fish are lower vertebrates adapted to the aquatic environment, so it is difficult to use them to model human space problems. Amphibians are similar to fish in that they are very dependent on water and, being anamniotes, are further away in morphology and metabolism from higher vertebrates. Mammals represent the optimal model for the possible extrapolation of results to humans, but their life support in a long orbital experiment is often too complicated and expensive.

Thus, in many ways, reptiles represent a compromise group, devoid of many weaknesses and possessing significant advantages (see Section 4) [81]. Geckos features make it unnecessary to create special incubators, thereby reducing the likelihood of egg damage and increasing the offspring’s chances of survival. As for the ability to attach to surfaces, young geckos have it almost immediately after hatching. At present, there are reasons to believe that the problem of providing thick-toed geckos with live food during space flights of more than 30 days is quite solvable [82]. Thus, it can be assumed that thick-toed geckos or some other species of reptiles may be promising model objects for studying reproduction and development in weightlessness. As it was rightly noted 27 years ago, a completely understandable and justified unwillingness to create a ‘zero-G zoo’ should not prevent us from setting tasks for which unusual model taxa may be needed and successfully solving these tasks [83].

So far, it has been established that the mating and fertilisation of eggs for some fishes and amphibian species can take place in space flights. Furthermore, some species of anamniotes born on space flight are able to live and reproduce after returning to Earth. The majority of embryos studied to date completed differentiation and morphogenesis. However, early stages of embryogenesis may be sensitive to the factors of space flight, including weightlessness. Abnormalities have however appeared during the early ontogeny for individuals in all classes of vertebrates studied in space flight conditions. The delay in the development of different systems, including the brain and sensory systems, was as a result of the spaceflight experiments with amphibia, birds and mammals. Postnatally reported alterations in dendritic sprouting and cortical synaptic patterns are also suggested as responses of the maturing nervous system to spaceflight conditions.

In general, the published data on the vertebrate early ontogenesis in space flight condition, including morphologal outcomes in nervous and sensory systems are incomplete and restricted. The avalible information evidence, that the development in microgravity can lead to many neurological alterations. Those can range from major and massive brain abnormalities to subtle delays in the development of the vestibular system and other reversible changes, which can be corrected for with further development.

There are also many other questions to be answered about vertebrate development under space flight conditions. Some of these include: does microgravity change embryonic cell migration? Are there alterations in surface tension and cell adhesion in embryo cells under microgravity? There is a deficit of developmental data on the cellular mechanisms underlying embryonic alterations induced by microgravity. More studies are warranted in whole organism behaviour, physiology, and morphology in microgravity [3], keeping in mind that organism plasticity allows many developmental alterations raised under the microgravity conditions to be compensated [84].

## Figures and Tables

**Table 1:** Chronological summary of space flight missions relating to vertebrate reproduction and development considered in the review.

Year	Species	Mission	Mission Duration	Age of Embryos and Foetuses (Larvae)	
				Launch	Fixation
1966	American grass (leopard) frog *Rana pipiens*	Gemini VIII mission	11 h	two-cell stage	in weightlessness cleavage stages: L + 40 h, L + 130 h, shortly before re-entry
1966	American grass (leopard) frog *Rana pipiens*	Gemini XII mission	3.9 days	two-cell stage	in weightlessness: L + 41 h, L + 85 h, several hours after recovery
1967	American grass (leopard) frog *Rana pipiens*	Biosatelllte II	2.5 days	two-cell stage	in weightlessness: L, L + 1 h, L + 2 h, L + 3 h (early cleavage), L + 32 h, L + 40 h (late gastrula), and L + 68 h (tail bud stage); the last two modules were to return live embryos
1971	Common frog *Rana temporaria*	Soyuz 10	2 days	Blastula—early gastrula	in weightlessness: early tail bud
1974	Zebrafish *Brachidanio rerio*	Soyuz 16	6 days	five somites	in weightlessness
1975	Clawed frog *Xenopus laevis*	Soyuz 17-Salyut 4	16 days	tail bud	in weightlessness
1975	Killifish *Fundulus heteroclitus*	Cosmos 782 satellite	19.5 days	fish eggs	after landing
1977	Zebrafish *Brachidanio rerio*	Soyuz 21–Salyut 5	9 days	late gastrula	in weightlessness
1976	Zebrafish *Brachidanio rerio*	Soyuz 22–Salyut 5	8 days	medium gastrula	on second day after landing
1977	Clawed frog *Xenopus laevis*	Soyuz 26–Salyut 6	20 days	early tail bud	in weightlessness
1979	Japanese quail *Coturnix japonica*	Cosmos-1129 satellite	18.5 days	fertilized eggs	only one of the quail embryos survived
1979	Brown rat *Rattus norvegicus*	Cosmos-1129 satellite	18.5 days	mating in space?	Not applicable
1980	Clawed frog *Xenopus laevis*	Soyuz 36–Salyut 6	8 days	mid neurula	on second day after landing
1981	Clawed frog *Xenopus laevis*	Soyuz 39–Salyut 6	9 days	early blastula	on second day after landing SD 45
1981	Clawed frog *Xenopus laevis*	Soyuz 40–Salyut 6	8 days	tail bud	on second day after landing SD 46
1983	Pregnant brown rat *Rattus norvegicus*	Cosmos-1514 satellite	4.5 days	13 gestational days	after landing: fetuses E18, pups—P0
1990	Japanese quail *Coturnix japonica*	Progress—MIR	16 days	fertilized eggs	in weightlessness: on days 4,7,10,14,16 of incubation; after landing: 4–5 days after hatching
1992	Clawed frog *Xenopus laevis*	STS-47 Spacelab SL-J	8 days	unfertilized eggs	in weightlessness: two-cell embryos, gastrulae, neurulae and swimming tadpoles
1992	Japanese quail *Coturnix japonica*	Soyuz TM-12—MIR	16 days	fertilized eggs	in weightlessness: on days 3, 7, 10 and 14 of incubation
1992–1993	Clawed frog *Xenopus laevis*	BION-10 satellite	11.5 days	SD 25	after landing: SD 47
1993	Clawed frog *Xenopus laevis*	STS-55 Spacelab D-2	10 days	SD 35	SD 45
1994	Medaka fish *Oryzias latipes*	STS-65 IML-2	15 days	mating and fertilization in space	after landing: hatching stage
1994	Japanese red-bellied newt *Cynops pyrrhogaster*	AstroNewt project STS-65–IML-2		embrios (at SD before the inner ear had formed and at the point just before the otoliths were formed) and fertilization in space	immediately after landing: a) SD-26–36 b) SD 8,9
1995	Japanese red-bellied newt *Cynops pyrrhogaster*	AstroNewt project unmanned SFU (Space Flyer Unit)		fertilization in space	after nine days from raising temperature
1994	Pregnant brown rat *Rattus norvegicus*	STS-66 NIH.Rodent 1	11 days	9 gestational days	after landing fetuses: E20, pups: P0
1995	Pregnant brown rat *Rattus norvegicus*	STS-70 NIH.Rodent 2	9 days	11 gestational days	after landing fetuses: E20, pups: P0
1995	Japanese quail *Coturnix japonica*	Progress 227–MIR STS-71 INCUBATOR 1	16 days	fertilized eggs	in weightlessness on days 7, 10, 14 and 17 of incubation
1995	Japanese quail *Coturnix japonica*	STS-71–MIR–STS-74 INCUBATOR 2	16 days	fertilized eggs	in weightlessness on days 7, 10, 14 and 16 of incubation
1996	*Rattus norvegicus*	STS-72 NIH.Rodent 3	9 days	pups: P5, 8, 14	after landing: P14,17,21
1996	Spanish ribbed newt *Pleurodeles waltl*	Fertile I Neurogenesis Mir Cassiopée expedition	16 days	fertilization in space	in weightlessness: different stages of the embryonic development. landing: hatching stages SD 31–32
1996	Japanese quail *Coturnix japonica*	STS-76–MIR–STS-79 INCUBATOR 3	16 days	fertilized eggs	in weightlessness on days 7, 10, 13 and 16 of incubation
1998	Rats pups *Rattus norvegicus*	NEUROLAB (STS-90)	16 days	pups: P8 and P14	after landing P24 and P30
1998	Pregnant mice *Mus musculus*	NEUROLAB (STS-90)	16 days		no data available
1998	Spanish ribbed newt *Pleurodeles waltl*	Fertile II (Mir Pégase expedition)	21 days	fertilization in space	in weightlessness: different stages of the embryonic development. landing: hatching stages SD 31–32
1999	Spanish ribbed newt *Pleurodeles waltl*	NEUROGENESIS (Mir Perseus expedition)	21 days	fertilization in space and different stages of the embryonic development (SD—0, 18, 43)	in weightlessness: different stages of the embryonic development; landing: swimming and feeding larvae SD 38, 39, 45
1999	Japanese quail *Coturnix japonica*	“Perepel CK-6” Soyus TM-28 MIR		13–14 days of incubation	hatching, early postnatal development
2001	Clawed frogs *Xenopus laevis*	French Soyuz taxi flight Andromède mission to the International Space Station	9.5 days	embryos SD 26–27 tadepoles—45	after landing: SD—46,47
2005	Thick-toed geckos (*Chondrodactylus turneri)*	Foton-M2	16 days	unfertilized eggs,	not applicable
2007	Thick-toed geckos (*Chondrodactylus turneri)*	Foton-M3	12 days	unfertilized eggs	not applicable
2010	Thick-toed geckos (*Chondrodactylus turneri)*	Bion-M1	30 days	unfertilized eggs	not applicable
2014	Ornate day geckos (*Phelsuma ornata*)	Foton-M4	44.5 days instead of the planned 60	not applicable	not applicable

L—launch, SD—stage of development, E—day of embryonal development, P—postnatal days.

## Data Availability

Not applicable.
